# Uma Nova Alternativa Terapêutica para o Tratamento da Hipertensão Pulmonar?

**DOI:** 10.36660/abc.20210935

**Published:** 2021-11-22

**Affiliations:** 1 Universidade de Buenos Aires Faculdade de Medicina Departamento de Patologia Buenos Aires Argentina Instituto de Fisiopatologia Cardiovascular, Departamento de Patologia, Faculdade de Medicina, Universidade de Buenos Aires, Buenos Aires – Argentina

**Keywords:** Doença Cardiopulmonar, Hipertensão Pulmonar, Transplante de Pulmão, Ratos, Monocrotalina, Insuficiência Cardíaca, Estresse Oxidativo, Cardiomegalia, Óleo de Copaiba, Fitoterapia, Fabaceae

Neste número da revista Arquivos Brasileiros de Cardiologia, foi publicada uma pesquisa importante e original sobre ratos.^1^ O trabalho é importante porque a hipertensão pulmonar representa uma patologia grave, de difícil tratamento e cuja única forma de aliviar o paciente é o transplante de pulmão com as dificuldades e limitações que esse difícil tratamento apresenta. O trabalho também é original porque muito se publicou sobre os efeitos benéficos do óleo de copaíba, por exemplo, sua ação antiinflamatória, seu efeito analgésico, bem como sua capacidade de prevenir infecções e melhorar o processo de cicatrização. No entanto, o efeito benéfico do óleo de copaíba na hipertensão pulmonar não foi descrito. Nesse sentido, o grupo liderado por Adriane Belló Klein, vem trabalhando há vários anos com o grande problema da hipertensão pulmonar em geral^2-4^ e os efeitos do óleo de copaíba nesta patologia,^5,6^ o que tornou este grupo de pesquisa uma referência internacional neste tema.

É importante mencionar que a hipertensão pulmonar induzida em ratos por monocrotalina produz lesões vasculares muito semelhantes às encontradas em pacientes com essa patologia.^7,8^ Em essência, o que mostra o trabalho publicado neste número da revista Arquivos Brasileiros de Cardiologia,^1^ é que a administração de aceite de copaíba a um grupo de ratas com hipertensão pulmonar induzida por monocrotalina, produz um claro efeito benéfico, por médio da diminuição dos indicadores em sangre periférica de stress oxidativo, uma redução da resistência vascular pulmonar, e um remodelamento favorável do ventrículo direito, com redução da hipertrofia, o que se traduz numa melhor função sistólica desse ventrículo. Este achado é muito importante porque é bem conhecido que a hipertrofia miocárdica é uma das principais causas de insuficiência cardíaca e morte súbita. A relação positiva entre o grau de hipertrofia e a diminuição do estresse oxidativo apresentada pelos autores é claramente comprovada neste trabalho pelo alto valor de r obtido quando essas duas variáveis foram correlacionadas. No entanto, e além desta importante correlação, estudos futuros devem determinar se o efeito benéfico no ventrículo direito se deve à redução do estresse oxidativo ou se há também um efeito direto do óleo de copaíba nas fibras miocárdicas. Talvez experimentos que pudessem ser feitos em corações isolados tornassem possível detectar ou não um efeito direto desse agente natural. Nesse sentido, é importante mencionar que os autores deste estudo também publicaram um interessante trabalho anteriormente^5^ onde mostraram que a administração do óleo de copaíba diminuiu os sinais de apoptose no ventrículo direito hipertrófico e atribuíram o melhor remodelamento a uma possível diminuição da já mencionada apoptose, obviamente esse mesmo mecanismo poderia estar agindo no trabalho que estamos discutindo agora. Porém, aqui é importante fazer alguns esclarecimentos que podem nos permitir entender melhor o que está acontecendo nesses corações com hipertrofia secundária à hipertensão pulmonar.

Em trabalho anterior, mostramos^9^ que a apoptose que se desenvolve durante a hipertrofia não se correlaciona com a função ventricular, ou seja, a apoptose não é necessariamente responsável pela diminuição da função ventricular como foi sugerido anteriormente, além disso, e ainda mais importante, a apoptose cardíaca não é apenas de miócitos, mas a grande maioria é devida a células não miócitos, o que inclui células endoteliais, fibroblastos ou macrófagos. Portanto, pode acontecer que a diminuição da apoptose por óleo de copaíba nesses corações com hipertrofia secundária à hipertensão pulmonar não seja principalmente nas células miocárdicas e, portanto, essa diminuição da apoptose não afeta a função ventricular, não estando envolvida no efeito benéfico do óleo de copaíba. Embora as variáveis relacionadas à apoptose não tenham sido medidas no presente trabalho, o fato de os mesmos autores terem sugerido em um trabalho anterior que esse mecanismo poderia ter sido incluído no efeito benéfico do óleo de copaíba, acho que justifica esse comentário sobre a apoptose. Ou seja, estamos comentando neste editorial um excelente trabalho experimental, bem desenhado, com resultados claros, importantes e originais, mostrando fortes evidências experimentais de um efeito benéfico do óleo de copaíba na inibição do estresse oxidativo. Talvez em trabalhos futuros outros mecanismos envolvidos possam ser descritos, que poderiam incluir, além do efeito do estresse oxidativo, uma ação direta sobre a fibra miocárdica ou um estudo mais detalhado da apoptose.
