# Supplementary material for: Uma Nova Alternativa Terapêutica para o Tratamento da Hipertensão Pulmonar?
Source: Arq Bras Cardiol. 2021 Nov 22;117(6):1113–4. [Article in Portuguese] doi: 10.36660/abc.20210935 (PMC8757148; doi:10.36660/abc.20210935)
Supplement: Translation

# Translation: A New Therapeutic Alternative for the Treatment of Pulmonary Hypertension?

**Affiliations:** 1 Buenos Aires Argentina Institute of Cardiovascular Physiopathology, Department of Pathology, Faculty of Medicine, University of Buenos Aires, Buenos Aires - Argentina

**Keywords:** Cor Pulmonale, Hypertension,Pulmonary, Lung Transplantation, Rats, Monocrotaline, Heart Failure, Oxidative Stress, Cardiomegaly, Copaiba Oil, Phytotherapy, Fabaceae

In this issue of the journal *Arquivos Brasileiros de Cardiologia,* important and original research work on rats is published.^1^ The work is important because pulmonary hypertension represents a serious pathology that is very difficult to treat. The only way to relieve patients is lung transplantation, but are very well known the difficulties and limitations of this treatment. The work is also original because a lot has been published about the beneficial effects of copaiba oil, such as its anti-inflammatory action, analgesic effect, and ability to prevent infections and improve the healing process. However, the beneficial effect of copaiba oil on pulmonary hypertension had not been described. In this sense, the group led by Adriane Belló Klein has been working for several years on this important issue of pulmonary hypertension in general,^2-4^ and the effects of copaiba oil on this particular pathology,^5,6^ which has made this research group an international benchmark on this theme.

It is important to mention that pulmonary hypertension induced in rats by monocrotaline produces vascular lesions very similar to those found in patients with this pathology.^7,8^ In essence, what the work published in this issue of the journal *Arquivos Brasileiros de Cardiologia*^1^ shows is that the administration of copaiba oil to a group of rats with pulmonary hypertension induced by monocrotaline produces a clear beneficial effect, through the decrease in peripheral blood indicators of oxidative stress, a reduction in pulmonary vascular resistance, and a favorable remodeling of the right ventricle, with a reduction in hypertrophy, which translates into a better systolic function of that ventricle. This finding is very important because it is well known that myocardial hypertrophy is one of the most important causes of heart failure and sudden death. The positive relationship between the degree of hypertrophy and the decrease in oxidative stress shown by the authors is clearly proven in this work by the high r-value obtained when these two variables were correlated. However, and beyond this important correlation, future studies should determine if the beneficial effect on the right ventricle is due to the reduction of oxidative stress or if there is also a direct effect of copaiba oil on myocardial fibers. Perhaps experiments that could be carried out on isolated hearts would make it possible to detect or not a direct impact of this natural agent. In this sense, it is important to mention that the authors of this study also published an interesting work previously^5^ where they showed that the administration of copaiba oil decreased apoptosis signals in the hypertrophic right ventricle and attributed the better remodeling to a possible decrease in the apoptosis mentioned above; obviously this same mechanism could be acting in the work that we are commenting now. Nevertheless, here it is important to make some clarifications that may allow a better understanding of what is happening in these hearts with hypertrophy secondary to pulmonary hypertension.

In previous work, we have shown^9^ that apoptosis that develops during hypertrophy does not correlate with ventricular function. That is, apoptosis is not necessarily responsible for the decrease in ventricular function as has been previously suggested, and in addition, and even more important, cardiac apoptosis is not only of myocytes, but the vast majority is due to nonmyocyte cells, which includes endothelial cells, fibroblasts, or macrophages. Therefore, it could be that the decrease in apoptosis due to copaiba oil in these hearts with hypertrophy secondary to pulmonary hypertension is not mainly in myocardial cells and therefore, this decrease in apoptosis does not affect ventricular function, not being involved in the beneficial effect of copaiba oil. Although variables related to apoptosis were not measured in the present work, the fact that the same authors have suggested in a previous work that this mechanism could have been included in the beneficial effect of copaiba oil, I think, justifies this comment on apoptosis. In other words, we are commenting in this editorial an excellent experimental work, well designed, with clear, important and original results, showing strong experimental evidence of a beneficial effect of copaiba oil in inhibiting oxidative stress. Perhaps in future works, other mechanisms involved could be described, which could include, in addition to the effect of oxidative stress, direct action on the myocardial fiber or a more detailed study of apoptosis.
